# Low Serum Alanine Aminotransferase Blood Activity Is Associated with Shortened Survival of Renal Cell Cancer Patients and Survivors: Retrospective Analysis of 1830 Patients

**DOI:** 10.3390/jcm13195960

**Published:** 2024-10-07

**Authors:** Menachem Laufer, Michal Sarfaty, Eyal Jacobi, Edward Itelman, Gad Segal, Maxim Perelman

**Affiliations:** 1 Department of Urology, Chaim Sheba Medical Center, Ramat Gan 5266202, Israel; 2 Faculty of Health and Medical Sciences, Tel-Aviv University, Tel-Aviv 6997801, Israel; 3 Institute of Oncology, Chaim Sheba Medical Center, Ramat Gan 5266202, Israel; 4 Faculty of Medicine, Ben-Gurion University, Beer-Sheva 8410501, Israel; 5 Cardiology Division, Rabin Medical Center, Beilenson Campus, Peta-Tiqva 4941492, Israel; 6 Department of Internal Medicine E, Rabin Medical Center, Beilenson Campus, Peta-Tiqva 4941492, Israel; 7 Education Authority, Chaim Sheba Medical Center, Ramat Gan 5266202, Israel; 8 Department of Internal Medicine “I”, Chaim Sheba Medical Center, Ramat Gan 5266202, Israel

**Keywords:** renal cell carcinoma, sarcopenia, frailty, alanine aminotransferase, survival, ALT

## Abstract

**Background:** Sarcopenia is characterized by a loss of muscle mass and function and is often associated with frailty, a syndrome linked to physical disability and shortened survival in various patient populations, including cancer patients. Low serum alanine aminotransferase (ALT) values, serving as a biomarker for sarcopenia, were previously associated with frailty and shortened survival in several cancers. In the current study, we aimed to test the association between low ALT and shorter survival in renal cell carcinoma (RCC) patients and survivors. **Methods:** This was a retrospective analysis of RCC patients and survivors, both in- and outpatients. We defined patients with sarcopenia as those presenting with ALT < 17 IU/L. **Results:** We identified records of 3012 RCC patients. The cohort included 1830 patients (mean age 65.6 ± 13.3 years, 68% were men) of whom only 179 underwent surgical treatment. Out of the eligible cohort, 811 patients (44.3%) had ALT < 17 IU/L, with a mean ALT value of patients within the low-ALT group of 11.79 IU/L, while the mean value in the higher ALT level group was 24.44 IU/L (*p* < 0.001). Patients in the low-ALT group were older (67.9 vs. 63.7 years; *p* < 0.001) and had lower BMIs (26.6 vs. 28; *p* < 0.001). In addition, patients with low ALT had lower hemoglobin values (12.14 vs. 12.91 g/dL; *p* < 0.001), higher serum creatinine (1.49 vs. 1.14; *p* < 0.001) and higher platelet to lymphocyte ratios (178 vs. 156; *p* < 0.001). In a univariate analysis, low ALT levels were associated with a 72% increase in mortality (95% CI 1.46–2.02, *p* < 0.001). In a multivariate model controlled for age, gender, hemoglobin, platelets, LDH, neutrophil to lymphocyte ratios and platelet to lymphocyte ratios, low ALT levels were still associated with a 27% increase in mortality (HR = 1.27, 95% CI 1.08–1.51; *p* = 0.005). **Conclusion.** Low ALT values, associated with sarcopenia and frailty, are also associated with shortened survival in RCC patients, and survivors and could potentially be applied for optimizing individual treatment decisions.

## 1. Background

### 1.1. Sarcopenia and Frailty Assessment amongst Cancer Patients

Assessment of patient’s physiological reserves is critical for treatment decision-making and the estimation of prognoses in cancer patients and survivors. Many cancer patients are surgical candidates, often necessitating major debilitating surgery. Therefore, the Charlson comorbidity index (CCI) and similar indices are commonly used before surgery in cancer patients [1]. Those tools are primarily objective but rather complicated, and other methods for a holistic estimation of patients’ overall capacity are subjective (at times, physicians just “eyeball” their patients) and are therefore difficult to define and standardize. ECOG performance status is a longstanding and universal feature oncologists use prior to medical therapy or radiation. It is short, easily understood and readily estimated. However, it is physician-assessed and, therefore, open to bias, potentially failing to integrate multimorbidity and frailty [2]. Sarcopenia is characterized by a loss of muscle mass and is correlated with frailty, a syndrome that leads to an increased likelihood of falls, disability, recurrent hospitalizations, and mortality [3,4,5]. In cancer patients, assessing sarcopenia and frailty improve the selection of patients eligible for major surgery and chemotherapy [6,7]. Both frailty and sarcopenia have been identified as markers for shortened survival in cancer patients [8].

### 1.2. Alanine Aminotransferase as a Biomarker for Sarcopenia and Frailty

Alanine aminotransferase (ALT) is a well-known intracellular enzyme, primarily in the liver parenchyma, that is commonly monitored in routine bloodwork. It catalyzes pyruvate to alanine in the skeletal muscle and alanine to pyruvate in the liver [9]. Elevated ALT blood levels are commonly used as a biomarker for hepatocellular injury; however, until recently, little was known regarding the clinical significance of lower-than-normal ALT. Several studies have demonstrated that a below-normal serum ALT activity, representing low muscle mass (sarcopenia, below expected for the same gender and age), is associated with shortened survival in older adults [10] and in patients hospitalized for various causes [11,12]. Furthermore, it was shown in several types of cancers that low ALT values are associated with increased frailty and shortened survival [13,14,15,16]. Based on our experience in previous studies, in diverse patient populations, we defined the value of 17 IU/L as a candidate value for differentiating frail from robust patients. Therefore, in the current study, we also used this value as a cutoff.

### 1.3. Renal Cell Carcinoma Patients and Survivors

RCC is the sixth most frequently diagnosed cancer in men and the 10th in women. The incidence of RCC has been increasing, and up to 17% of patients suffer from distant metastases at the time of diagnosis. Well-established risk factors include older age, smoking, obesity, hypertension, and chronic kidney disease [17,18]. Treatment options for RCC have changed dramatically over the past two decades. Surgery, specifically a radical nephrectomy or nephron-sparing surgery, is the mainstay of non-metastatic disease. However, active surveillance of small masses and tumor ablation are viable alternatives. The introduction of immune checkpoint inhibitors has significantly improved the overall survival of advanced RCC patients. Immune checkpoint inhibitor doublets or, when administered in combination with a vascular endothelial growth factor, tyrosine kinase inhibitors have (VEGFR-TKI) become the standard primary therapy in metastatic disease. Recently, immune checkpoint inhibitors have been used to offer neoadjuvant and adjuvant treatment settings in patients undergoing surgery [19]. Prognostic models have been developed for localized disease based on tumor stage, grade, subtype, and performance status. However, there is a poor level of evidence regarding their routine use. Traditional risk group assessment for patients with metastatic RCC also use performance statuses and some blood tests: low hemoglobin, high calcium and high LDH blood levels are well-established factors that help determine patients’ risk group classifications. Newly recognized prognostic markers such as platelet counts, white blood cell subtypes and platelet to lymphocyte ratios probably represent inflammatory responses to the cancer. Newer markers in patients receiving targeted treatments are used routinely but have limited accuracy [20]. Despite being a disease frequently found in older and often frail adults, there is only a small amount of available data in regard to guiding treatment decisions in those patients with metastatic RCC. The recent approval of many new agents for this disease poses a clinical challenge: how to best utilize these drugs in a population otherwise under-represented in clinical trials [21,22]. A few studies have investigated more accurate parameters, including sarcopenia and frailty in RCC patients. Unsurprisingly, they found that both syndromes correlated with surgical and oncological outcomes [23,24,25,26]. Both need to be assessed at any stage of RCC to define the most suitable treatment strategy, ranging from surveillance to aggressive treatment [27,28,29].

### 1.4. Aim of the Current Study

In the current study, we assessed the association between the low serum activity of ALT, suggestive of sarcopenia and frailty, and shorter survival in a large cohort of renal cell carcinoma patients and survivors.

## 2. Methods

### 2.1. Study Cohort

In the cohort featured in the current study, we included all men and women diagnosed with RCC who were treated in a large, tertiary medical center as outpatients or inpatients during the years 2013 to 2022. Patients underwent either surgery, immunotherapy, targeted therapy, or active surveillance. The study was conducted according to the guidelines of the Declaration of Helsinki and approved by the Institutional Review Board of the Chaim Sheba Medical Center (IRB approval # SMC-24-1252-D) on 13 May 2024. Following this approval and a waiver of informed consent in light of the retrospective nature of this study, all relevant patient characteristics, demographics, and clinical data were retrieved from the patients’ electronic medical records. We excluded patients with ALT activity levels higher than 40 IU that are generally associated with injured liver cells in various types of hepatitis and, therefore, not a reliable marker for striated muscle mass. The final cohort includes patients with ALT levels that were established at the time of RCC diagnosis. The primary outcome of the current study was all-cause mortality. Survival data were available for all subjects from the National Population Registry.

### 2.2. Statistical Analysis of Data

The normality of the distribution of continuous variables was determined using the Anderson–Darling and Shapiro–Wilk tests. If normally distributed, continuous variables were expressed as mean ± standard deviation (SD) and, if skewed, by median with the interquartile range (IQR). Categorical variables were presented as numbers and percentages (N; %). Continuous data were compared with the student’s *t*-test, and categorical data were compared using chi-square or Fisher exact tests. A log-rank test was used to analyze survival, later depicted using a Kaplan–Meier curve. Univariate cox regression modeling was used to determine the unadjusted Hazard Ratio (HR) for the primary outcome, and a multivariate model was constructed to examine the correlation and control for possible confounders. An association was considered statistically significant for a two-sided *p* value of less than 0.05. All analyses were performed using R software version 4.1.0 (R Foundation for Statistical Computing, Boston, MA.).

The normality of the distribution of continuous variables was determined using the Anderson–Darling and Shapiro–Wilk tests. If normally distributed, continuous variables were expressed as mean ± standard deviation (SD) and, if skewed, by median with the interquartile range (IQR). Categorical variables were presented as numbers and percentages (N; %). Continuous data were compared with the student’s *t*-test, and categorical data were compared using chi-square or Fisher exact tests. A log-rank test was used to analyze survival, later depicted using a Kaplan–Meier curve. Univariate cox regression modeling was used to determine the unadjusted Hazard Ratio (HR) for the primary outcome, and a multivariate model was constructed to examine the correlation and control for possible confounders. An association was considered statistically significant for a two-sided *p* value of less than 0.05. All analyses were performed using R software version 4.1.0 (R Foundation for Statistical Computing, Boston, MA.).

## 3. Results

**Figure 1:**
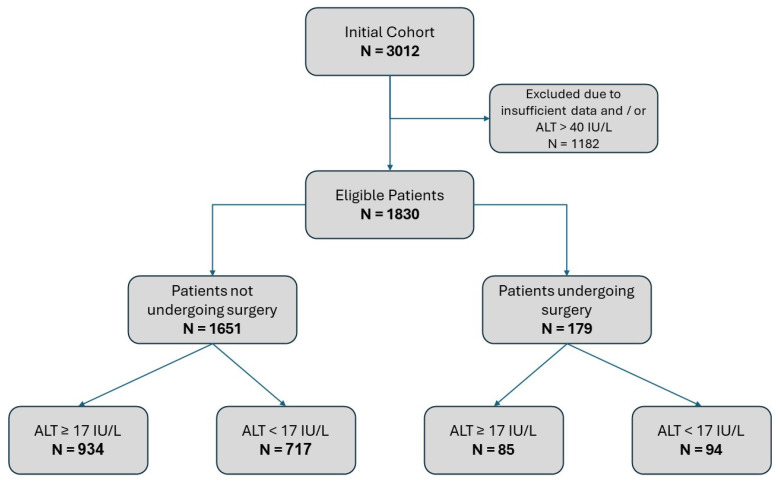
Consort flow diagram of study patients.

A total of 3012 renal cell carcinoma patients’ records were identified; after applying our exclusion criteria (available epidemiological data and ALT levels within the normal range), the final study population included 1830 patients. We identified 179 patients who underwent surgical resection (either a radical or partial nephrectomy). Within the whole eligible patients’ cohort, 811 (44.3%) had ALT activity levels that were lower than 17 IU/L and, therefore, compatible with our definition of patients at high risk of suffering from sarcopenia and frailty. Figure 1 details patient consort flow and exclusion diagrams.

The mean age for the entire cohort was 65.3 ± 13.3 years. Relevant patient demographics and clinical characteristics are detailed, according to their ALT levels (lower or ≥ 17 IU/L), in Table 1: patients in the low-ALT group were, as expected, older (67.9 ± 13.5 vs. 63.7 ± 12.9 years, *p* < 0.001), had lower body mass index (BMI) values (26.6 ± 4.9 vs. 28 ± 5.1, *p* < 0.001) and had higher percentages of diabetes mellitus (26% vs. 22%, *p* = 0.036), arterial hypertension (57% vs. 51%, *p* = 0.017) and atrial fibrillation (9.5% vs. 6.1%, *p* = 0.008). They also had statistically significant lower values of hemoglobin, higher values of blood creatinine and a higher ratio of platelet to lymphocyte counts. Patients in the lower ALT group had a significantly shorter time in regard to survival and/or loss of follow-up (1822 ± 1487 vs. 2045 ± 1457 days, *p* = 0.001).

### 3.1. Univariate Analysis

**Figure 2:**
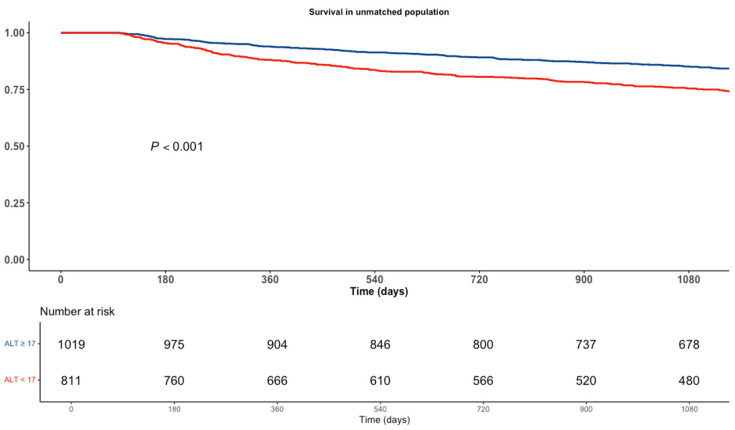
Kaplan–Meir Survival Analysis according to ALT levels.

In a univariate analysis, low ALT levels were associated with a significant 72% increase in mortality (95% CI 1.46–2.02, *p* < 0.001). Figure 2 shows a Kaplan–Meir curve for the crude survival analysis according to the ALT levels.

### 3.2. Multivariate Analysis

In a multivariate model (Table 2), low ALT levels were still associated with a 27% increased risk of mortality (HR = 1.27, 95% CI 1.08–1.51, *p* = 0.005).

## 4. Discussion

### 4.1. Personalized vs. Precision Medicine for Cancer Patients

The realms of advanced diagnostics and therapeutics in patients suffering from RCC have significantly advanced during the past several years. Nevertheless, most advancements were in the domains of precision medicine [30,31], addressing diseased tissue itself, rather than in the domain of personalized medicine, that is, addressing the patient rather than the disease. Moreover, some authors describe the movement from precision to personalized medicine in the sense of deepening our understanding of the molecular structure of the cancerous tissue [32], with some authors even bundling both precision and personalized medicine together, namely, PPM [33]. The authors of the current manuscript defy this concept, seeing personalized medicine as taking a “zoom-out” from the diseased tissue to the whole gestalt of a patient. We concentrate on personalized medicine in terms of sarcopenia and frailty assessment.

In former studies, we established the association between low ALT and worse clinical outcomes in other patient populations suffering from different, hematologic and solid malignancies: myelodysplastic syndrome [13], chronic lymphocytic leukemia [16], prostate adenocarcinoma [14] and bladder cancer [15].

### 4.2. Sarcopenia and Frailty of RCC Patients

As presented at the forefront of this article, sarcopenia, and frailty are a pillar of morbidity in cancer patients as a whole and in RCC patients precisely [25,28]. Massaad et al. evaluated the relative effects of different patients’ characteristics on clinical outcomes among post-operative patients with RCC metastasis. Amongst other variables, sarcopenia and frailty were also assessed [34]. Sarcopenia was assessed by measuring the L3 skeletal muscle index (L3-SMI) on axial CT images at the level of lumbar L3 vertebra, while frailty was assessed using the modified frailty index. Their results found no significant association between these measures and patients’ overall survival. We hypothesize that these findings stem from the fact that all patients were already metastatic as opposed to our cohort of patients with metastatic and localized disease. Moreover, although strongly validated, their methods for sarcopenia and frailty assessment were rather complex.

Low ALT assessment as part of personalized medicine offered for RCC patients: Similarly to our groups’ findings in patients with other urinary system malignancies (urinary, bladder and prostate), we showed that low ALT blood activity, representing a low total-body striated muscle mass, is associated with a poor prognosis. Relying on previous findings that confirmed the association of low ALT with sarcopenia and frailty, we similarly ascertained in RCC patients that low ALT is a reliable biomarker for sarcopenia and frailty.

Our study population was rather diverse, featuring both RCC patients with/without surgical treatment and RCC survivors. This was deliberate, as we strove to give clinicians patient stratification tools that would be effective for as wide a patient population as possible. Low ALT was not significantly associated with clinical outcomes in the whole population, and we would have tested it in RCC sub-populations; however, this was not the case. Future studies may follow, and sub-populations of RCC patients will be investigated.

## 5. Conclusions

Low ALT blood levels, measured in RCC patients and survivors, are associated with sarcopenia and frailty. This finding should be assimilated into the scheme of personalized medicine for RCC patients as part of their future plans for surgery, medical therapy, and prognostication.

## 6. Limitations

This was a single-center, retrospective study; therefore, our conclusions necessitate further verification in larger, multi-centered, preferably prospective studies.

## Figures and Tables

**Table 1:** Patients’ characteristics according to ALT serum activity level.

	Whole Study Cohort N = 1830	ALT ≥ 17 IU/L N = 1019	ALT < 17 IU/L N = 811	*p* Value
ALT (Mean IU/L [SD])	18.83 (8.01)	24.44 (6.07)	11.79 (3.04)	<0.001
**Patients’ Demographics**				
Age (years, Mean [SD])	65.6 (13.3)	63.7 (12.9)	67.9 (13.5)	<0.001
Male Gender (N [%])	1238 (68)	704 (69)	534 (66)	0.155
BMI (Mean [SD])	27.4 (5)	28 (5.1)	26.6 (4.9)	<0.001
**Clinical Background**				
Diabetes Mellitus (N [%])	428 (23)	219 (22)	209 (26)	0.036
Dyslipidemia (N [%])	657 (36)	379 (37)	278 (34)	0.214
Arterial Hypertension (N [%])	980 (54)	520 (51)	460 (57)	0.017
COPD (N [%])	113 (6)	53 (5.2)	60 (7.4)	0.065
CHF (N [%])	67 (3.7)	30 (2.9)	37 (4.6)	0.088
Atrial Fib. (N [%])	139 (8)	62 (6.1)	77 (9.5)	0.008
S/P Stroke (N [%])	95 (5.2)	47 (4.6)	48 (5.9)	0.252
**Laboratory Parameters**				
Albumin (g/dL, Mean [SD])	3.9 (1.38)	3.93 (0.58)	3.86 (1.97)	0.32
Hemoglobin (g/dL, Mean [SD])	12.57 (2.02)	12.91 (1.9)	12.14 (2.09)	<0.001
Platelets (K/μL, Mean [SD])	236 (95)	233 (91)	239 (100)	0.17
Neut./Lymph. (Mean [SD])	4.87 (5.85)	4.67 (5.06)	5.12 (6.71)	0.106
Plt./Lymph. (Mean [SD])	166 (118)	156 (115)	178 (121)	<0.001
LDH (IU/L, Mean [SD])	243 (149)	245 (159)	240 (135)	0.496
Calcium (mg/dL, Mean [SD])	7.69 (3.43)	7.56 (3.56)	7.85 (3.26)	0.069
Creatinine (mg/dL, Mean [SD])	1.29 (1.08)	1.14 (0.69)	1.49 (1.4)	<0.001
Time to Death/End of Follow-up (Days, Mean [SD])	1946 (1474)	2045 (1457)	1822 (1487)	0.001

**Table 2:** Multivariate Analysis.

Patient Characteristics	HR (95% CI)	*p* Value
ALT < 17 IU/L	1.27 [1.08–1.51]	0.005
Age	1.05 [1.05–1.06]	<0.001
Male gender	1.6 [1.33–1.92]	<0.001
Hemoglobin (g/dL)	0.84 [0.81–0.87]	<0.001
Platelets (K/mcl)	1.00 [1.00–1.00]	0.003
LHD (IU/L)	1.00 [1.00–1.00]	<0.001
Platelets/Lymphocytes	1.00 [1.00–1.00]	0.007

## Data Availability

Research data associated with this research will be available from the corresponding author upon request.

## References

[1] Sinha P., Kallogjeri D., Piccirillo J.F. (2014). Assessment of comorbidities in surgical oncology outcomes. J. Surg. Oncol..

[2] Simcock R., Wright J. (2020). Beyond Performance Status. Clin. Oncol..

[3] Cruz-Jentoft A.J., Sayer A.A. (2019). Sarcopenia. Lancet.

[4] Cruz-Jentoft A.J., Bahat G., Bauer J., Boirie Y., Bruyère O., Cederholm T., Zamboni M. (2019). Sarcopenia: Revised European consensus on definition and diagnosis. Age Ageing.

[5] Picca A., Coelho-Junior H.J., Calvani R., Marzetti E., Vetrano D.L. (2022). Biomarkers shared by frailty and sarcopenia in older adults: A systematic review and meta-analysis. Ageing Res. Rev..

[6] Korc-Grodzicki B., Downey R.J., Shahrokni A., Kingham T.P., Patel S.G., Audisio R.A. (2014). Surgical Considerations in Older Adults with Cancer. J. Clin. Oncol..

[7] Ryan A.M., Prado C.M., Sullivan E.S., Power D.G., Daly L.E. (2019). Effects of weight loss and sarcopenia on response to chemotherapy, quality of life, and survival. Nutrition.

[8] Au P.C.-M., Li H.-L., Lee G.K.-Y., Li G.H.-Y., Chan M., Cheung B.M.-Y., Wong I.C.-K., Lee V.H.-F., Mok J., Yip B.H.-K. (2021). Sarcopenia and mortality in cancer: A meta-analysis. Osteoporos. Sarcopenia.

[9] Liu Z., Que S., Xu J., Peng T. (2014). Alanine Aminotransferase-Old Biomarker and New Concept: A Review. Int. J. Med. Sci..

[10] Ramaty E., Maor E., Peltz-Sinvani N., Brom A., Grinfeld A., Kivity S., Segev S., Sidi Y., Kessler T., Sela B. (2014). Low ALT blood levels predict long-term all-cause mortality among adults. A historical prospective cohort study. Eur. J. Intern. Med..

[11] Ruhl C.E., Everhart J.E. (2013). The Association of Low Serum Alanine Aminotransferase Activity with Mortality in the US Population. Am. J. Epidemiol..

[12] Segev A., Itelman E., Avaky C., Negru L., Shenhav-Saltzman G., Grupper A., Segal G. (2020). Low ALT Levels Associated with Poor Out-comes in 8700 Hospitalized Heart Failure Patients. J. Clin. Med..

[13] Uliel N.B., Segal G., Perri A., Turpashvili N., Lerner R.K., Itelman E. (2023). Low ALT, a marker of sarcopenia and frailty, is associated with shortened survival amongst myelodysplastic syndrome patients: A retrospective study. Medicine.

[14] Laufer M., Perelman M., Sarfaty M., Itelman E., Segal G. (2023). Low Alanine Aminotransferase, as a Marker of Sarcopenia and Frailty, Is Associated with Shorter Survival Among Prostate Cancer Patients and Survivors. A Retrospective Cohort Analysis of 4064 Patients. Eur. Urol. Open Sci..

[15] Laufer M., Perelman M., Segal G., Sarfaty M., Itelman E. (2023). Low Alanine Aminotransferase as a Marker for Sarcopenia and Frailty, Is Associated with Decreased Survival of Bladder Cancer Patients and Survivors-A Retrospective Data Analysis of 3075 Patients. Cancers.

[16] Hellou T., Dumanis G., Badarna A., Segal G. (2023). Low Alanine-Aminotransferase Blood Activity Is Associated with Increased Mor-tality in Chronic Lymphocytic Leukemia Patients: A Retrospective Cohort Study of 716 Patients. Cancers.

[17] Capitanio U., Bensalah K., Bex A., Boorjian S.A., Bray F., Coleman J., Russo P. (2019). Epidemiology of Renal Cell Carcinoma. Eur. Urol..

[18] Bukavina L., Bensalah K., Bray F., Carlo M., Challacombe B., Karam J.A., Kassouf W., Mitchell T., Montironi R., O’Brien T. (2022). Epidemiology of Renal Cell Carcinoma: 2022 Update. Eur. Urol..

[19] Chen Y.W., Wang L., Panian J., Dhanji S., Derweesh I., Rose B., McKay R.R. (2023). Treatment Landscape of Renal Cell Carcinoma. Curr. Treat. Options Oncol..

[20] Klatte T., Rossi S.H., Stewart G.D. (2018). Prognostic factors and prognostic models for renal cell carcinoma: A literature review. World J. Urol..

[21] Maia M.C., Adashek J., Bergerot P., Almeida L., dos Santos S.F., Pal S.K. (2018). Current systemic therapies for metastatic renal cell car-cinoma in older adults: A comprehensive review. J. Geriatr. Oncol..

[22] Esther J., Hale P., Hahn A.W., Agarwal N., Maughan B.L. (2019). Treatment Decisions for Metastatic Clear Cell Renal Cell Carcinoma in Older Patients: The Role of TKIs and Immune Checkpoint Inhibitors. Drugs Aging.

[23] Ueki H., Hara T., Okamura Y., Bando Y., Terakawa T., Furukawa J., Harada K., Nakano Y., Fujisawa M. (2022). Association between sarcopenia based on psoas muscle index and the response to nivolumab in metastatic renal cell carcinoma: A retrospective study. Investig. Clin. Urol..

[24] Noguchi G., Kawahara T., Kobayashi K., Tsutsumi S., Ohtake S., Osaka K., Umemoto S., Nakaigawa N., Uemura H., Kishida T. (2020). A lower psoas muscle volume was associated with a higher rate of recurrence in male clear cell renal cell carcinoma. PLoS ONE.

[25] Ishihara H., Nishimura K., Ikeda T., Fukuda H., Yoshida K., Iizuka J., Kondo T., Takagi T. (2024). Impact of body composition on outcomes of immune checkpoint inhibitor combination therapy in patients with previously untreated advanced renal cell carcinoma. Urol. Oncol. Semin. Orig. Investig..

[26] Rosiello G., Larcher A., Fallara G., Cignoli D., Re C., Martini A., Capitanio U. (2023). A comprehensive assessment of frailty status on surgical, functional and oncologic outcomes in patients treated with partial nephrectomy—A large, retrospective, single-center study. Urol. Oncol. Semin. Orig. Investig..

[27] Courcier J., De La Taille A., Lassau N., Ingels A. (2021). Comorbidity and frailty assessment in renal cell carcinoma patients. World J. Urol..

[28] Campi R., Berni A., Amparore D., Bertolo R., Capitanio U., Carbonara U., Erdem S., Ingels A., Kara O., Klatte T. (2022). Impact of frailty on perioperative and oncologic outcomes in patients undergoing surgery or ablation for renal cancer: A systematic review. Minerva Urol. Nephrol..

[29] Walach M.T., Wunderle M.F., Haertel N., Mühlbauer J.K., Kowalewski K.F., Wagener N., Rathmann N., Kriegmair M.C. (2021). Frailty predicts outcome of partial nephrectomy and guides treatment decision towards active surveillance and tumor ablation. World J. Urol..

[30] Sharma R., Kannourakis G., Prithviraj P., Ahmed N. (2022). Precision Medicine: An Optimal Approach to Patient Care in Renal Cell Carcinoma. Front. Med..

[31] Massari F., Santoni M., Di Nunno V., Cimadamore A., Battelli N., Scarpelli M., Cheng M., Lopez-Beltran A., Cheng L., Montironi R. (2018). Quick steps toward precision medicine in renal cell carcinoma. Expert Rev. Precis. Med. Drug Dev..

[32] Gambardella V., Tarazona N., Cejalvo J.M., Lombardi P., Huerta M., Roselló S., Fleitas T., Roda D., Cervantes A. (2020). Personalized Medicine: Recent Progress in Cancer Therapy. Cancers.

[33] Krzyszczyk P., Acevedo A., Davidoff E.J., Timmins L.M., Marrero-Berrios I., Patel M., White C., Lowe C., Sherba J.J., Hartmanshenn C. (2018). The growing role of precision and personalized medicine for cancer treatment. Technology.

[34] Massaad E., Saylor P.J., Hadzipasic M., Kiapour A., Oh K., Schwab J.H., Shin J.H. (2021). The effectiveness of systemic therapies after surgery for metastatic renal cell carcinoma to the spine: A propensity analysis controlling for sarcopenia, frailty, and nutrition. J. Neurosurg. Spine.

